# Commercially Available Essential Oil Formulas as Repellents Against the Stored-Product Pest *Alphitobius diaperinus*

**DOI:** 10.3390/insects10040096

**Published:** 2019-04-01

**Authors:** Jacek Francikowski, Bartosz Baran, Mikołaj Cup, Jakub Janiec, Michał Krzyżowski

**Affiliations:** 1 Department of Animal Physiology and Ecotoxicology, Faculty of Biology and Environmental Protection, University of Silesia, Bankowa 9, 40-007 Katowice, Poland; bartosz.m.baran@gmail.com (B.B.); michal.krzyzowski@o2.pl (M.K.); 2 College of Inter-Faculty Individual Studies in Mathematics and Natural Science, University of Warsaw, Stefana Banacha 2C, 02-097 Warsaw, Poland; mkicup@gmail.com; 3 Faculty of Physics, University of Warsaw, Pasteura 5, 02-093 Warsaw, Poland; jakub.janiec@me.com

**Keywords:** lesser mealworm, essential oils, repellency, spatial preference, locomotor activity

## Abstract

The main aim of the presented paper is to assess the potential repellent effect of selected essential oils (EOs) against the lesser mealworm (*Alphitobius diaperinus*), which can cause economic losses in storage and in the poultry industry. Due to the development of pesticide resistance in *A. diaperinus* populations, as well as an attempt to limit extensive use of potentially harmful pesticides in food-related industries, there is a strong need for the development of alternative methods of dealing with *A. diaperinus* infestations. Because of their cost-effectiveness, availability and low vertebrate toxicity, EOs are promising agents in pest management. In the presented paper four off-the-shelf EOs: mint, vanilla, lemon and citronella (and mixtures of them) were tested as potential repellents. Moreover, a novel preference assay, providing an extended analysis of the preference and the locomotor response, was used. The most effective EOs were: citronella and lemon. EOs mixtures were generally more repellent than individual EOs, with the lemon and vanilla 1:1 mixture acting as the strongest repellent. A few of the tested EOs caused significant alterations to the locomotor activity, although no direct relation was observed. In conclusion, EOs can be potentially used as repellent agents in *A. diaperinus* management. Additionally, data on the locomotor activity may lead to designing better push-pull strategies in pest management.

## 1. Introduction

The lesser mealworm, *Alphitobius diaperinus* (Panzer) is a cosmopolitan insect pest of stored products and the poultry industry. It infests stored grain and other amylaceous products [1]. Moreover, it is a potential vector of several pathogens and parasites [2]. Due to the lack of chitinase enzyme in some broiler strains, *A. diaperinus*, once ingested, may cause bowel obstruction leading to microscopic perforations in the intestinal wall of birds [3]. Additionally, a large-sized *A. diaperinus* population can cause structural damage to buildings, especially in their thermal insulation, which leads to a drastic increase in heating costs of buildings [4].

Insecticide resistance was reported in numerous populations of *A. diaperinus* [5,6]. Those reports, along with growing concerns over the extensive use of synthetic pesticides in food production [7], show the urgency to develop a new approach to the protection against *A. diaperinus*. Essential oils (EOs) are particularly promising in meeting the needs as they are: plant-derived, easily biodegradable [8], widely accessible, considered safe for vertebrates (including humans) and, above all, effective both as insecticides and repellents [9,10]. Suitability of the EO-based formulas as insecticides against *A. diaperinus* is proven [11]. The information concerning the repellent potential of the EOs against *A. diaperinus* may be crucial in the development of the push-pull management systems [12]. The recent research focused on answering the question whether (and how strong) the presence of EOs in the air repels *A. diaperinus*. A novel, non-pitfall, preference (expressed as preference index—PI) test was used to assess the highly developed exploratory behaviour of *A. diaperinus* [13].

The most commonly used assays to measure the olfactory preference are the Y maze [14,15,16] test and the pitfall test [17,18,19]. The pitfall test is doubtlessly the easiest assay to assemble, and, to a great extent, it resembles the properties of the traps used in field practice [20]. The Y maze is a standard for assaying the choice making in various animals. Both assays rarely provide continuous monitoring of the movement of insects during the test. Moreover, after the decision is made, an insect is either trapped in a pitfall or removed from the setup (the Y maze assay), thus it cannot change the choice. Such approaches do not take into account the exploratory behaviour that may lead to random choices, therefore introducing noise to the data. The procedure described in [13] addresses the issue by allowing the insect to explore the test chambers freely, while its position is continuously tracked. Such an approach, based on easily accessible hardware and free software, can improve the quality of the data obtained on the olfactory preference and, simultaneously, provide additional information describing the movement parameters (speed, distance travelled, etc.) which may indicate a general physiological response to the tested substances.

In the presented paper, four commercially available essential oils were tested. They were tested separately for each oil and in mixtures to assess the potential synergistic effect which was observed in numerous experiments on microorganisms and insects [21,22,23]. Using the synergistic effect in creating insecticide formulas may significantly reduce the required amount of substances, thus their costs.

## 2. Materials and Methods

### 2.1. Essential Oils

The essential oils used in the experiment were obtained from local vendors (vanilla, lemon, mint EOs from Meister Oil, Poland and citronella EO from Vera, Stanisławów Drugi, Poland).

The oils were tested separately and as mixtures. The mixtures were formulated by mixing equal parts of the tested EOs. For each oil and mixture, a series of dilutions was prepared in ultrapure deionised water of the following concentrations: 0.001%, 0.01%, 0.1%, 1% and 10% (*v*/*v*). The obtained suspensions were stirred until emulsified and poured into the respective bubbler before phase separation occurred. The bubbler, controlling the airstream, was filled with an equal volume of ultrapure water.

### 2.2. Insects

The insects were reared in boxes with coconut fibre bedding in stable conditions: temperature of 30 °C, relative humidity of 50% and the photoperiodic regime of 12/12 h LD. The insects had access to water and food (standard dog food pellets, Pedigree, McLean, VI, USA) *ad libitum*. In all the experiments, imagoes of *A. diaperinus* (Panzer) of both sexes at the average age of 30 days were used.

### 2.3. Behavioural Test

**Figure 1:**
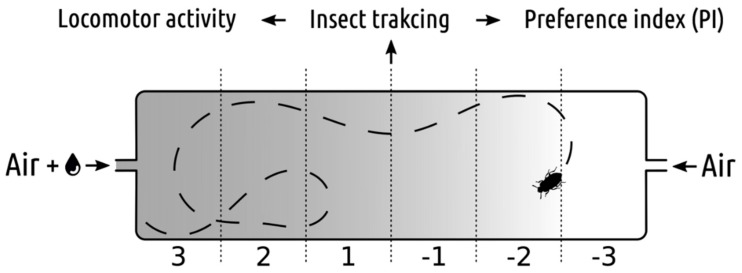
Schema of experimental chamber with air inputs (with or without EOs) and zones values for preference index (PI) quantification.

Behavioural tests were conducted using the setup described in the article by Baran et al. (2018) [13] (Figure 1). Forty-eight individuals were used for a single concentration of an essential oil or a tested mixture. Each insect was placed separately in a rectangular chamber made of clear Lucite, with tubes attached to both ends. The tubes supplied a constant flow of humidified air from one end and humidified air with the tested odour from the other [13]. The airflow was kept at 10 L/h. The inlet air was pumped through a bubbler containing a mineral oil (to capture possible contaminants from the pump) and then was separated into either a water bubbler or a bubbler with an aqueous solution of the odour compound. The constant, homogeneous background light was provided with a red transilluminator placed underneath. The insects were able to explore the chambers freely. Recordings of experimental procedure were captured with Microsoft LifeCam 500 webcam and VirtualDub 1.10.4 software as .avi files. Recordings lasted 20 min at the framerate of 15 fps and the resolution of 640 × 860 px. The whole setup was placed in an enclosed, ventilated box isolated from external visual and acoustic stimuli.

Tracking and analyzing the movements of the insects was conducted in the same way as in the article by Baran (2018) [13]. Three parameters were calculated for all the analyzed videos: preference index (PI), distance and resting time. Preference index was calculated as the total number of frames with the insect in each compartment of the experimental chamber. The distance and the resting time are the two basic locomotor activity parameters which indicate how active the insect was during the experiment. The parameters are expressed as a pathway length in pixels and as the percentage of the time spent resting. The 20-min long videos were analyzed as two separate 10-min-long intervals (interval I and II) because of the difference in the behavioural reaction of the insects. In the first interval, activity and exploration level were very high whereas in the second interval the locomotor activity and the exploration activity were much lower.

### 2.4. Statistical Analysis

Statistical analyses were conducted with Statistica^®^ software v10 (Dell Software, Aliso Viejo, CA, USA). Data distribution normality was verified with the Shapiro-Wilk test, which concluded that most of the variables were not normally distributed. The groups were compared using the non-parametric Kruskal-Wallis test (Kruskal-Wallis ANOVA) with the median test. For all the tests *p* < 0.05 was applied. For multiple comparisons, Bonferroni correction was used. Figures were prepared with use of GraphPad Prism version 6.00 for Windows (GraphPad Software, La Jolla, CA, USA).

## 3. Results

### 3.1. Behavioural Effects—Single Oils

#### 3.1.1. Mint EO

**Figure 2:**
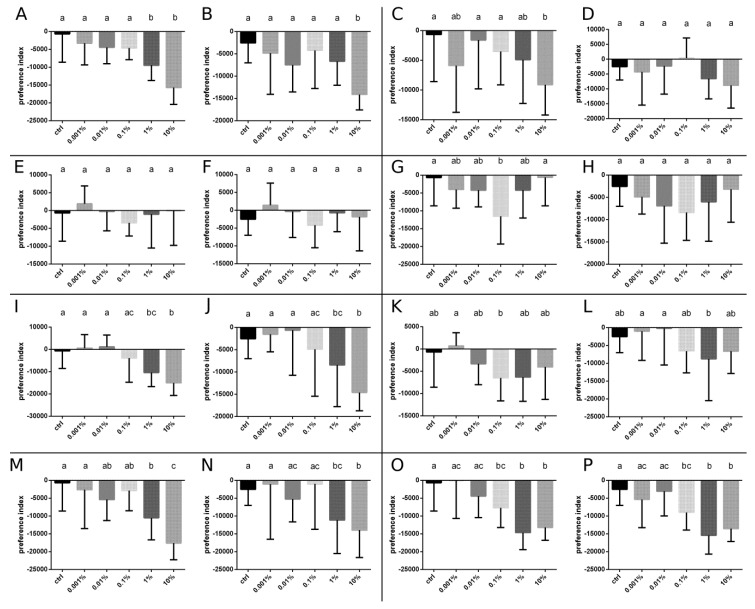
Preference index (PI) for insects treated with a single essential oil or a mixture (1:1 or 1:1:1) of essential oils in different concentrations and the control. Median and quartiles are presented, Kruskal-Wallis test *p* < 0.05. Different letters indicate statistically different groups. Different letters indicate statistically different groups. Citronella EO (**A**) interval 1, (**B**) interval 2; lemon EO (**C**) interval 1, (**D**) interval 2; mint EO (**E**) interval 1, (**F**) interval 2; vanilla EO (**G**) interval 1, (**H**) interval 2; 1:1 mix of citronella and lemon EOs (**I**) interval 1, (**J**) interval 2; 1:1 mix of citronella and vanilla EOs (**K**) interval 1, (**L**) interval 2; 1:1 mix of vanilla and lemon EOs (**M**) interval 1, (**N**) interval 2; 1:1:1 mix of vanilla, citronella and lemon EOs (**O**) interval 1, (**P**) interval 2.

**Figure 3:**
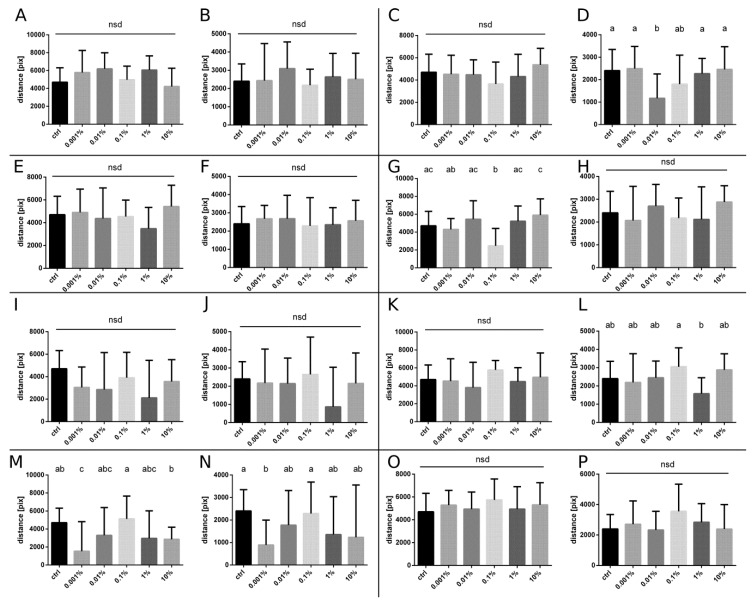
Distance (px) reached for insects treated with a single essential oil or a mixture (1:1 or 1:1:1) of essential oils in different concentrations and the control. Median and quartiles are presented, Kruskal-Wallis test *p* < 0.05. Different letters indicate statistically different groups. Different letters indicate statistically different groups. Citronella EO (**A**) interval 1, (**B**) interval 2; lemon EO (**C**) interval 1, (**D**) interval 2; mint EO (**E**) interval 1, (**F**) interval 2; vanilla EO (**G**) interval 1, (**H**) interval 2; 1:1 mix of citronella and lemon EOs (**I**) interval 1, (**J**) interval 2; 1:1 mix of citronella and vanilla EOs (**K**) interval 1, (**L**) interval 2; 1:1 mix of vanilla and lemon EOs (**M**) interval 1, (**N**) interval 2; 1:1:1 mix of vanilla, citronella and lemon EOs (**O**) interval 1, (**P**) interval 2.

**Figure 4:**
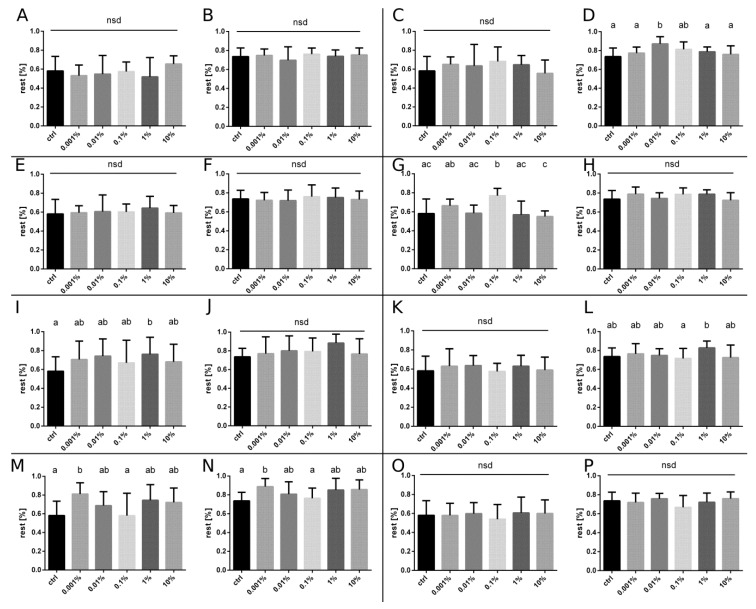
Resting time (%) for insects treated with a single essential oil or a mixture (1:1 or 1:1:1) of essential oils in different concentrations and the control. Median and quartiles are presented, Kruskal-Wallis test *p* < 0.05. Different letters indicate statistically different groups. Citronella EO (**A**) interval 1, (**B**) interval 2; lemon EO (**C**) interval 1, (**D**) interval 2; mint EO (**E**) interval 1, (**F**) interval 2; vanilla EO (**G**) interval 1, (**H**) interval 2; 1:1 mix of citronella and lemon EOs (**I**) interval 1, (**J**) interval 2; 1:1 mix of citronella and vanilla EOs K interval 1, (**L**) interval 2; 1:1 mix of vanilla and lemon EOs (**M**) interval 1, (**N**) interval 2; 1:1:1 mix of vanilla, citronella and lemon EOs (**O**) interval 1, (**P**) interval 2.

No significant effects were observed, PI indicated neither repellent nor attractive properties (Figure 2). The locomotor activity parameters did not differ from the control group (Figure 3 and Figure 4).

#### 3.1.2. Lemon EO

Significant repellency was observed (Figure 2) at the highest concentration (10%) and it was distinguishable in both of the 10-min intervals. Moreover, considering the locomotor activity, a twofold decrease in the total travelled distance was observed (Figure 3) at the concentration of 0.01% in the second interval. Simultaneously, for the same concentration, the percentage of the total time spent resting (Figure 4) was significantly higher.

#### 3.1.3. Citronella EO

Citronella EO showed the strongest repellent effect among all the tested single EOs. The repellent effect was observed in both intervals. The repellency increased with the concentration (1%, 10%) in the first interval. In the second interval, the observed repellency was also significant but the relation to the concentration differed. Only the effect of the highest concentration was statistically significant (Figure 2). The locomotor parameters did not differ significantly in comparison to the control group (Figure 3 and Figure 4).

#### 3.1.4. Vanilla EO

The statistically significant effect was observed only in the first interval—the repellency at the concentration of 0.1%. It was the lowest effective concentration of the single oil in the experiment. At higher concentrations (1%, 10%) the effect was not observed, thus the Pi/concentration curve for vanilla EO is U-shaped. Despite the absence of any significant effects in the second interval, the same U-shaped curve is apparent (Figure 2). The effects on the locomotor activity level are observable only in the first interval. At the concentration of 0.1%, the distance covered was much shorter and almost twice as much time was spent resting (Figure 3 and Figure 4).

### 3.2. Behavioural Effects—Oil Mixtures

#### 3.2.1. Citronella/Vanilla EOs

A significant effect on the PI appeared only in the second interval, at the concentration of 1%. In comparison to the results obtained while using single OEs, a highly repellent effect of either citronella EO or vanilla EO was suppressed in the mixture in the first interval. The effect observed in the second interval could be compared to the effects of the single EOs present in the mixture, as follows: U-shaped PI curve of vanilla EO and significant repellency in the second interval, the same as for citronella EO (Figure 2). There were not any significant effects on the locomotor activity in comparison to the control group (Figure 3 and Figure 4).

#### 3.2.2. Lemon/Vanilla EOs

In both intervals, a significant repellency of the mixture was observed at the concentrations of 1% and 10% (Figure 2). Even the lowest concentration (0.001%) had a significant effect on the locomotor activity, decreasing the distance travelled and increasing the resting time (Figure 3 and Figure 4).

#### 3.2.3. Citronella/Lemon EOs

A significant repellent effect was observed for the concentrations of 1% and 10% in both intervals. In comparison to the single EOs, a substantial amplification of the effectiveness was noticeable. The repellent effect of the mixture occurred at a lower concentration than for lemon oil and lasted longer than for both oils tested separately (Figure 2).

#### 3.2.4. Citronella/Vanilla/Lemon EOs

In the first interval, repellency was observed at the concentrations of 1% and 10%. The lowest concentrations were the most effective. In the second interval, the repellent effect was observable at the concentration of between 0.1% and 10% (Figure 2). Despite the presence of noticeable effects on the spatial preference, there were no significant changes in the locomotion pattern (Figure 3 and Figure 4).

## 4. Discussion

For many years, researchers have considered essential oils as potential pesticides against stored-product pests [9,24,25,26]. Many of them demonstrate insecticidal properties against common pests such as beetles. However, most studies focus on the direct effect of the oils–mortality. Results presented in the manuscript prove that EOs may also act as effective repellents. Moreover, it is the first report on the effectiveness of vanilla essential oil against *A. diaperinus*.

Out of all the tested EOs, only mint EO did not affect insect behaviour at all. Similar results were also observed in the experiment on common house mosquito (*Culex pipiens*) [27]. Out of all the tested EOs, peppermint oil showed the weakest repellent effect. Such a result may result from the relatively low toxicity of the main constituent of the mint oil, menthol (Isman). Vanilla, citronella and lemon EOs showed significant repellency which occurred mainly at higher concentrations (0.1–10%) with the only exception of vanilla EO which effectively repelled *A. diaperinus* only at 0.1%. The strongest repellency (lowest effective concentration) was observed for citronella EO. The lowest (yet significant) repellency for single oils was observed for lemon.

Similar results were reported by [28]. In the experiment, four essential oils were used (*Citrus limonum, Litsea cubeba, Cinnamomum cassia and Allium sativum* L.) and, out of all the tested substances, mint was proved to have the weakest influence on the *A. diaperinus*. Data from the assessment conducted on *Tribolium castaneum* beetles show citronella EO is an effective repellent (65% insects repelled), however, in the particular study, the insects were directly exposed to the tested EOs, which could affect the observed performance [29]. Moreover, citronella EO was reported to be an effective repellent against *A. aegypti* with the repellent action lasting for up to two hours, however it was tested by applying undiluted oil directly on the human skin (trongtokit). Regrettably, more comprehensive comparison of results obtained on citronella EO to other studies is difficult due to highly variable periods of tests utilized by different authors–usually, the repellency is measured hours or even days after the application of the tested EOs.

Similarly, the concentrations often start at 1%, and in the presented study, there were significant effects observed at 0.1% or 0.01%. The analysis of both the preference index and the spatial behaviour as the aspects of the response of insects to EOs was considered to provide a more comprehensive overview of the potential repellent. The obtained results showed a large discrepancy at the level of both responses. The repellent effect was not explicitly linked with the decreased locomotor activity. In single concentrations of vanilla EO (interval I, 0.1%) and lemon EO (interval II, 0.01%), the reduction in the distance travelled, as well as the prolongation of resting time, were observed. Low concentrations of active compounds might trigger immobility while higher concentrations evoked the escape response [10,30], which was manifested as the increased locomotor activity and shortened immobility time. It was an important observation which would be impossible without implementing a multiparameter analysis.

The behavioural changes, which were concentration- and time-dependent were not as pronounced as expected, given the PI value, especially at the higher concentrations of the used essential oils and their mixtures. The most distinct changes occurred mainly at the lower concentrations. It suggests that either the received dose of the repellent was not high enough to cause changes at the physiological level or the transition to the sections of lower concentrations of the essential oil allowed to maintain activity at a constant level. Changes in the distance level were visible only in lemon (interval II, 0.01%), vanilla (interval I, 0.1%), citronella- vanilla mix (interval II, 0.1 / 1%), vanilla- lemon (interval I and II, 0.001%). The percentage of the activity time shows the relationship between the results for different concentrations and the level of the distance covered. If an insect walked a longer distance, it was characterized by the shorter rest period. The only exception where the relationship was not observed was the citronella and lemon mix (1% interval I) for which the distance did not differ statistically from the control, unlike the rest time.

There are studies showing the influence of essential oils on the behaviour of insects, not only at the purely sensory level, i.e., changes in the behaviour to avoid the stimulus, but also at the level of direct impact on the nervous system [9,31,32]. Potentially, two sites of influence are indicated: acetylcholinergic and octopaminergic systems. The articles suggest the possible effects of selected components of essential oils on the receptors of acetylcholine and octopamine. They are two neurotransmitters which modulate the level of the locomotor activity in insects [33]. Thus, disorders in their reception may form the basis for the observed changes. There are also visible changes in the level of cAMP, which clearly correlate with the level of the locomotor activity [34]. Such a relation is evident, especially for the methylxanthines such as caffeine, i.e., another group of sub-agents modulating the level of the locomotor activity. At higher concentrations, the tested components of essential oils could lead to a decrease, instead of an increase, in the cAMP concentration, which might indicate their antagonistic effect on the receptors. Of course, changes in the behaviour resulting from the physical irritation, damage to the structures of the respiratory or sensory systems cannot be excluded. It can be assumed that the groups with changes in the locomotor activity may be those where the insects were exposed to the concentrations that caused the physiological effect but were not sufficiently repellent at the sensory level. The article also presents the results of using a mixture of previously tested essential oils. Many available papers report increased efficiency of mixtures of substances, in comparison with solutions containing single compounds [10,28]. The secondary principle of the experiment was to investigate the potential additive effect caused by the interaction of the active ingredients of previously described essential oils. The obtained mixtures potentially increased the chance of interactions between the components of all the used oils. The effect on the spatial behaviour profoundly differed between the single oils and the mixtures. A strong additive repellent effect was observed in the mixture containing lemon, citronella and vanilla, as well as in lemon and citronella EOs.

Nevertheless, the starkest example of the additive effect was observed in the citrus and vanilla EOs. Both citrus and vanilla oils, separately, are weak repellents, and citrus oil is the weakest repellent among the tested oils. However, in the mixture with vanilla oil, citrus oil has the highest PI among all the tested oils and the mixtures (at the concentration of 10%). As such, it could be considered to be a co-operative evidence of EOs, which is a well-recognized mode of the EO action [14,23]. In the context, vanilla itself can be regarded as a potential synergist among the repellent agents. Studies on vanillin (the main constituent of the extracts from *Vanilla* sp.) show that it is as a potent synergistic agent against *Aedes aegypti*. It was reported to increase efficiency significantly when used in the mixtures with the EOs (in particular in 1/1 *v*/*w* proportions, similar to those tested in the presented study) [19].

Moreover, the addition of vanillin significantly increased the duration of effect of the tested mixtures. In vitro biochemical studies showed that vanillin alone is also antagonistic to the octopamine receptor in American cockroach (*Periplaneta americana*) [34]. In the presented context, the data obtained from the lemon and vanilla mixture are highly coherent with the data available in the literature.

Large-scale use of insect repellents is commonly incorporated in the integrated management strategy, consisting of planned placement of lures and repellents, hence it is named the push-pull approach. Effectiveness of the push-pull pest management relies on modifying the spatial behaviour of insects. We suggest that the general exploratory ability should be considered together with the preference of visiting a particular area. The ability of a specific substance to downregulate or upregulate the exploratory behaviour of insects should be considered as a critical variable in designing the whole pull-push setup. Terpenes contained in EOs can interact with various receptors that impact the baseline behavioural activity [35]. Moreover, alterations to the exploratory behaviour may affect exposure to the EO-based insecticides, therefore considering behavioural parameters may be crucial in designing efficient insecticidal agents.

## 5. Conclusions

Considering the PI, citronella EO has the strongest action at the concentration of 10%, while among the 1:1 mixes the strongest one is vanilla with citronella (the most potent effect in the entire experiment was observed at the concentration of 10%). The blend of three EOs (vanilla, citrus, and citronella), at the concentration of 10%, had the effect (15,000 PI) comparable with the most effective EOs and mixtures. The lowest concentrations at which significant effects appeared were 0.1 and 1% for vanilla and citronella EOs, respectively. In the case of the blend of three EOs, the decrease of the lowest effective concentration along with the increase in the repellent effect was observed. Such a result was unique in comparison to the other mixes.

Therefore, most of the tested essential oils and their mixes may be considered to be a potential way to manage *A. diaperinus* infestations. Additionally, the results of the assessments show the potential for the use of the mixtures of essential oils to manage stored-product pest infestations, due to the clearly observed additive effect. Moreover, according to the data obtained from the conducted assessments, there was no direct relation between the locomotor activity and the strength of the repellent.
